# Challenges of foot self-care in older people: a qualitative focus-group study

**DOI:** 10.1186/s13047-019-0315-4

**Published:** 2019-01-18

**Authors:** Maija Miikkola, Tella Lantta, Riitta Suhonen, Minna Stolt

**Affiliations:** 1 Department of Nursing Science, University of Turku, Turku, Finland; 2 Turku University Hospital, Turku, Finland; 3 City of Turku, Welfare Division, Turku, Finland

**Keywords:** Foot self-care, Foot health, Older people, Prevention, Focus-group interviews, Qualitative research

## Abstract

**Background:**

Foot health is an important aspect of general health, and it can be maintained and promoted through foot self-care. However, little is known about older people’s experiences of caring for their feet. The aim of this study was to gather knowledge about experiences of foot self-care from the perspective of healthy older people in order to improve their welfare and their management of foot health.

**Methods:**

A qualitative descriptive design with focus groups was used. Seventeen older people recruited from daytime activity centres participated in the focus groups (*n* = 4). The data were analysed using inductive content analysis.

**Results:**

The participants described their foot self-care as including various activities, but they were hindered by the following factors: physical (e.g. changes in nail structure), external (e.g. seeking help from multi-level professionals) and internal (e.g. related to ageing). Foot self-care was considered to be important, but it was not systematically carried out. The participants thought that health-care professionals neglected patients’ feet.

**Conclusions:**

Older people use a variety of methods to care for their feet. However, several factors hinder their ability to do so. Older people need advice, education and support to maintain their foot health. Future research is needed to identify effective ways to support older people in foot self-care and improve their welfare as active citizens.

## Background

Proper foot self-care is commonly described as including nail and skin care, washing and drying the feet each day, doing foot exercises, and wearing socks and shoes that fit and are made of appropriate materials. Even small actions have an impact on foot health [1]. Taking care of the feet, either personally or by regularly visiting a podiatrist, can prevent foot problems. Moisturising the feet keeps the skin elastic [2] and prevents skin deformities (e.g. corns and calluses) [3]. Hosiery can contribute to shock absorbency [4], and foot exercises promote balance and ankle flexibility [5]. Shoes provide balance and security when walking [6]. Foot problems, especially pain, can have a detrimental effect on quality of life [7, 8]. Many people are not aware of the importance of foot self-care and the related practices, and this can be enhanced by education [9, 10]. Without care, minor foot problems can easily become major problems that require podiatric care [11]. Physical difficulties, inadequate knowledge and poor perceived importance of foot self-care have been identified as barriers to foot self-care practices in older people with diabetes [12].

Older people experience a considerable number of foot problems [13, 14]; every third older person has at least one foot problem [15], with women being more likely to suffer [16, 17]. Populations all over the world are ageing [18], and strategies for active ageing and prevention are supported in national [19] and international guidelines [20]. There is an emphasis on people taking control of their own health and adopting lifestyle changes that may reduce the possibility of their developing such diseases as cancer and diabetes. Ageing affects foot health [21, 22] and is associated with changes in foot characteristics [22]. However, ageing is not the only cause of poor foot health, foot pain and the loss of foot-related functional ability [23]. For example, female sex, and pain in other bodily regions associate with foot health [23]. Foot self-care can be beneficial in preventing foot problems; therefore, it can promote older people’s wellbeing and their ability to manage at home [1, 24, 25]. Maintaining foot health by caring for one’s feet and choosing suitable footwear can also prevent falls [26, 27]. Poor footwear has been found to contribute to falls in older people [27, 28].

Self-management and self-care are part of health promotion. Advancing health and well-being, promoting health and particularly foot health, is important to support active ageing [20]. Although, foot health among older people has been widely researched, still little is known about older people’s foot self-care, especially among older people who do not have long-term diseases that affect the feet, such as diabetes, rheumatoid arthritis or psoriasis. Thus, this study explored foot self-care among older people by asking the following research questions: what kind of foot self-care do older people practise, and which factors are associated with older people’s foot self-care? Gathering the perspectives of older people is necessary in order to find out whether they want to invest in foot health and what issues need to be emphasised in general practice, for example. In this study, older people are defined as people aged 65 or over [29].

## Methods

### Aim

The aim of this study was to gather knowledge about experiences of foot self-care from the perspective of healthy older people in order to improve their welfare and their management of foot health.

### Research ethics

The responsible conduct for research indicated in the Finnish Advisory Board’s Guidelines on Research Integrity [30] was followed, and ethical approval was obtained from the university’s Ethics Committee (16/2013). Permission to conduct the study (12755–2013, decision number 9/10) was obtained from the Welfare Division of the cooperating municipality in which the daytime activity centres were located. Permission to use the organisation’s space for interviews was obtained from the centres. The participants received verbal and written information about the study during recruitment and before interviewing. All participants signed a written informed consent form and could withdraw from the study at any time. In the written informed consent form, demographic variables, such as name and birth year, were collected; addresses were collected only if the participant wished to receive a transcript of their focus group.

### Design, setting and participants

To elicit personal experiences, a qualitative descriptive method [31] using focus-group interviews was selected for this study. Focus groups were guided by a moderator (MM) and followed by co-researcher (TL). Focus groups can generate in-depth knowledge from people by combining certain features [32] of those who give their views about foot self-care. Moreover, it provides insights into how people think, captures deeper information of the phenomena under investigation and facilitates understandings of the factors influencing behavior [32]. Consecutive sampling was used [33], and the sample was taken from one small municipality (with a population of 250,000) in western Finland. The participants were recruited from three daytime activity centres. Activities offered at the centres included exercise classes, leisure activities, and health and welfare counselling for older people. In total, the activity centres received nearly 60,000 visits per year. This setting was chosen because visitors to these types of centres represent an active section of the ageing population, and in this study the purpose was to capture the experiences of these citizens.

Foot care in Finland is provided by registered podiatrists who have Bachelor’s degree. Podiatry and foot care services are available in public and private health care organizations. Public podiatric care is tax-based and focused strongly in patients with diabetic risk foot. The access to public podiatric care requires a referral from a physician, public health nurse or diabetes nurse [34]. Podiatrists work independently, still collaborating with physicians and other health care professionals. Nursing homes have podiatrists or visiting podiatrists who deliver foot care to older patients. Nurses working in the nursing homes support older people with their daily foot hygiene and skin care. Moreover, several private podiatry clinics offer wide range of services for all citizens; however, the patients cover the costs by themselves. In addition, foot care services in private sector are offered also by beauticians and foot care assistants.

### Recruitment

Consecutive sampling was used to recruit older people (aged 65 and older) who were living in their own home and did not have long-term foot-related diseases. The first author and cooperating members of staff at the centres approached potential participants at random in public spaces where older people spent time. Information about the study and its purpose was provided, and voluntary participation was requested. Research advertisements were also displayed in the centres. Individuals were excluded if they were younger than 65, had diseases that required special foot self-care, or had diabetes, rheumatoid arthritis or psoriasis. The goal was to recruit older people who did not have long-term systemic health problems: those whose foot self-care is important but seldom studied. Those who fulfilled the inclusion criteria and were willing to participate were recruited and given a written information sheet. At that time, only names and telephone numbers were collected from the potential participants. Potential participants were either given a place and time for a preliminary interview during the recruitment process or contacted later by phone to schedule an interview time.

### Data collection

The focus groups were held in February and March 2014 in two daytime activity centres in rooms appointed by staff at the cooperating centres. The focus groups lasted from 37 min to 47 min and were recorded on tape. The focus groups were conducted by the first author (MM) and a co-author (TL). The co-author was responsible for the tape recorder, taking field notes and ensuring all the relevant research topics were covered. Field notes were made during the interviews in case the tape recording failed. In addition, the field notes were analysed to evaluate the data saturation, which was done by both interviewers [32]. Saturation was achieved in the third focus group; this was confirmed in the fourth focus group, as the participants produced no new relevant information about the topics.

The focus-group interview guide (Table 1) were based on previous studies [15, 35–37]. The themes included basic good and correct practice of foot self-care; practising foot self-care; and associated factors, such as motivational issues and managing foot self-care. To stimulate the conversation, the participants were asked an introductory question about what they considered to be foot self-care. The participants were encouraged to speak broadly about the themes, and questions were asked and modified based on the issues that the participants took into consideration. Before the data collection began, a pilot focus group was conducted with three older people. The focus-group themes were covered and the participants provided in-depth responses. No changes to the focus-group themes were made. Therefore, the data that resulted from the pilot focus group were included in the final data.

**Table 1:** Focus group questions (interview guide)

Questions:	
1. What foot self-care activities do older people perform?	
- How do you care for your own feet?	
- When do you care for your feet?	
2. What factors are associated with performing foot self-care in older people?	
- How do you manage with foot self-care?	
- What motivates you to care for your own feet?	
- What hampers caring for your own feet?	

### Data analysis

The transcribed data were analysed using inductive content analysis (Table 1) to identify and organise the text by categorising and abstracting it in relation to the research questions [38]. The focus groups were transcribed verbatim. Only the manifest content of the focus groups was used for the analysis [38], which means that during the analysis no hidden meanings were sought behind, for example, the laughter of the participants. To avoid misinterpreting the participants’ linguistic expressions, latent content was not analysed. The transcribed materials were read several times to form an overall impression of the data. After reading, all sentences and phrases that related to the research questions were entered into tables in order to organise the data (Table 2). No labelling was done at that point. After all the relevant sentences had been organised, answers to the research questions were sought from the material. The tables were read again; sentences and phrases about the same thing, such as participants’ habits and features, were grouped into different tables. Category names and codes were created based on the content of the sentences and condensed into smaller units, taking care to avoid losing the meaning of the words. The condensed category names were then reduced to an abstraction. After this, the abstractions were labelled with codes of words. Codes that shared characteristics were grouped into sub-categories, which were given names that described the codes. The sub-categories that could be combined were labelled with suitable category names. The category names and codes were reflected on and discussed with the co-authors. Two main themes emerged from the research questions, and the categories were finally combined under the names of the two main themes.

**Table 2:** Example of analysis process from category name to theme

Category name	Condensed category name	Abstraction of condensed category name	Code	Sub-category	Category	Theme
*N11: Well during wintertime skin dries out much more and heels become drier – you must moisturise them all the time.*	During winter skin and heels are drier and moisturising is needed all the time	More foot cream in winter when skin is dry	More foot care during winter	Seasonal changes	External factors	Factors associated with foot self-care
*R4: Well, unfortunately it is still quite modest, although it wouldn’t take more than fifteen minutes so I don’t understand why. Laziness.*	(Foot care) is still modest; although it takes only fifteen minutes it’s laziness	Modest foot care because of laziness, even though time spent is minimal	Modest foot care because of laziness	Personality factors	Internal factors	

## Results

### Characteristics of the participants

Seventeen participants participated in four focus groups. The target was to have four to eight participants per group [31–33]; however, due to timetable challenges, the focus groups were conducted with three to five people. Four men and thirteen women participated to focus groups. They were born between 1924 and 1948: the youngest participant was 66 and the oldest was 90 (mean 77). Five focus groups were scheduled; however, one was cancelled due to a lack of participants. No other demographic data were collected.

**Fig. 1:**
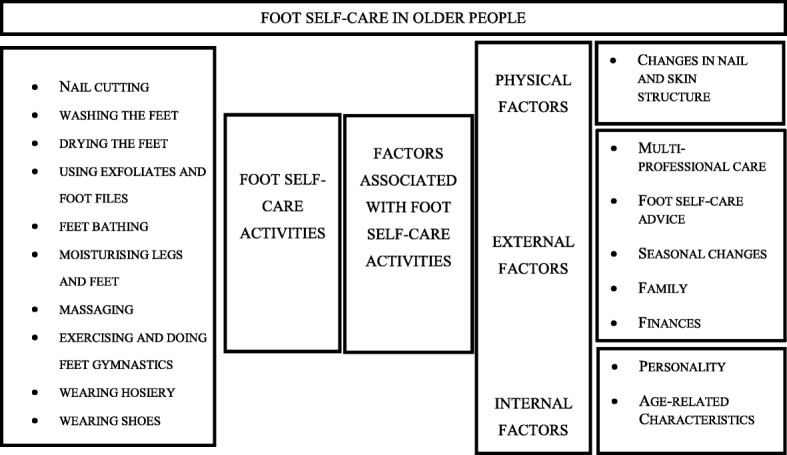
Foot self-care in older people based on an inductive content analysis

Based on the analysis, two major themes on foot self-care in older people were found: foot self-care activities and factors associated with foot self-care activities in older people (Fig. 1). Older people described not only diverse foot-self-care activities that they felt to be important, but also diverse factors associated with foot self-care.

### Foot self-care activities

Older people described the practice of foot self-care as including various activities: 1) nail cutting; 2) washing the feet; 3) drying the feet; 4) using exfoliators and foot files; 5) soaking the feet in water; 6) moisturising the legs and feet; 7) massaging the feet; 8) exercising the feet; 9) wearing hosiery; and 10) wearing shoes. Some described the activities only, and others understood foot self-care as part of their overall health and hygiene care. Based on the focus groups, foot self-care for older people was characterised by the identification when professional foot care was needed.

R8: well, self-care is applying foot cream after washing and cutting your nails. That’s all.

Nail-cutting was done mostly by the participants. They also highlighted that they contacted professionals to support them in nail-cutting (such as beautician, foot care assistant, podiatrist). Feet were washed only once or twice a week. Feet washing was seldom mentioned as a separate activity, instead it was seen part of daily hygiene tasks, such as having a shower. A few participants mentioned drying the feet. Some used exfoliators and foot files to remove dry skin. Some used footbaths for problems such as corns and calluses.

The participants rarely moisturised using foot cream, and only a few applied foot cream every day. Taking care of the rest of the body was prioritised over foot care. The participants did not want to do excessive washing because they thought that it would dry their skin. Dry skin motivated moisturising, and some massaged their feet at the same time to keep them warm. Blood circulation was also connected with wearing shoes – and especially socks – indoors to avoid the feet freezing.

R3: I moisturise twice a week. I know I should do it more but I haven’t had any problems. You wake up when something happens.

Most of the participants exercised their feet regularly, and they had a comprehensive understanding of the importance of moving and exercising. They felt that all exercise, including walking, was important for continuing active ageing. The participants had received advice and tips on exercising from magazines, exercise groups and health-care professionals. The need for exercise was widely acknowledged among the participants.

R4: If you stay where you are, that’s where you’ll stay. I feel that you must move, even if it hurts a little.

The participants did not have established views about materials for hosiery and socks. The word “socks” was used in a general way to describe ankle socks and stockings. The participants mentioned liking soft and warm socks. Participants who wore compression socks enjoyed the support, although they felt cold during the wintertime. Shoes were important for maintaining movement. Suitable shoes were described using several terms: comfortable, adjustable, right-sized, fitting and roomy. The participants said they sometimes bought a larger shoe size in order to have enough width for broad feet. They thought that shoe size did not change with ageing; however, they understood the individual needs of feet and shoe choices. The participants were aware of the relationship between shoes worn at an early age and foot problems in later life.

R7: Isn’t it true that they say shoes should always be good, that you shouldn’t wear ill-fitting shoes? They’re not good for the feet.

The participants faced difficulties finding shoes for special needs, such as larger feet or foot orthoses. In addition, participants with no specific foot issues also found buying shoes a stressful experience. When choosing shoes, comfort was mostly more important than price or fashion. Most of the participants did not wear shoes indoors, as they felt that wool or other warm socks were better. The participants were afraid of stumbling if wearing shoes indoors.

### Factors associated with foot self-care activities in older people

Based on the descriptions provided by older people, three overarching factors associated with older people’s foot self-care was found: 1) physical, 2) external and 3) internal.

### Physical factors

#### Changes in nail and skin structure

This was the main physical factor that caused difficulties in foot self-care. One of these changes was thickening of the nails, which was considered to be age-related. Nail-cutting was one of the most difficult self-care activities for participants. Stiffness of the back, old age and gaining weight impeded the nail-cutting position. Nail-cutting was also the main reason for seeking help for foot care.

R6: It’s somehow hard. Nails get thicker when you age. Some sort of handy technique should be found for when scissors are not enough.

The participants had noticed changes in their skin and that their skin had dried as they got older. Changes in the nail and skin structure, such as corns, calluses and ingrown nails, were problematic for participants. Some treated these foot problems; for example, with a knife or a pedicure machine, which can be hazardous when not used by a professional.

### External factors

#### Multi-professional care

Thickened toenails and other foot problems were the main reason for seeking help: multi-professional care was a major factor in the participants’ foot self-care. Some trusted their foot care entirely to professional care, some regularly visited a pedicurist or other foot-care professional, and only a few did all their foot care themselves. However, professional foot care was not seen as part of their overall health care but more as an indulgence and pleasure. Some of the participants felt that they needed to visit a specialist, but had not yet done so.

The participants tried to take care of their feet independently for as long as they could. Treatment and professional care was sought when pain and limitations in daily life occurred or when their problems seemed too severe to handle by themselves. If needed, professional help was requested from foot-care specialists or physicians. Nevertheless, the participants felt that too little attention had been paid to their feet. Feet were not noticed by professionals without the participants asking specific foot-related questions. The participants used common sense when evaluating the need for help.

R9: Well doctors don’t, they should – take the feet into consideration, but it’s not considered nowadays. It should be that all parts of the body are checked.

#### Advice on foot self-care

Participants sought advice about foot-care products and self-care for foot problems from, for example, the pharmacy and specialist health-care stores. Some participants were not sure where to ask for advice. Products for foot self-care were also difficult to find, and the participants had not received any help or advice about finding suitable products. The participants noted that with proper nail clippers they could cut thickened nails. Some bought products such as foot cream from a pharmacy, supermarket or a pedicurist, and some followed the recommendations of others. Even though the participants were willing to ask for advice, they found it difficult to know which product to buy. They did not use the Internet to search for information, and they did not considered it to be a reliable source of information.

R2: I could check on the Internet, but I don’t trust that kind of thing. I don’t trust it. There’s this and that – it must be something you can rely on.

#### Seasonal changes

The participants saw associations between seasonal changes and their foot self-care. In Finland, the climate is very different in summer and winter. The participants noticed that during summer their skin, especially the heels, is dry. However, some needed more foot care during winter. Walking barefoot and wearing summer shoes were considered to have a negative effect on the condition of the feet.

#### Family

Participants had not asked for help with foot care from their family; nor had they thought of asking. Asking was not considered to be appropriate; they did not feel that they would get the help, even if they asked. Participants preferred to pay for professional foot care rather than ask for help from relatives, because professionals had the appropriate training and skills. There was a feeling among the participants that although hands could be shown to others, the feet should be kept hidden. Feet were seen as more intimate.

R10: I wouldn’t ask for help, I would go to a specialist – as long as you can do something by yourself and there are experts it’s enough for me. But you never know when you get older.

#### Finances

Foot self-care activities and visiting multi-level foot-care specialists were seen as expensive necessities. However, some gladly paid the price and did everything that was needed for their health, including their foot health. Visiting a foot-care specialist was seen as an expensive but valuable investment for older people. The price was the only negative aspect. If foot problems occurred, the participants were willing to reduce spending on other things (e.g. leisure activities or buying clothes) to pay for treatment. The participants were willing to seek help from private health-care providers if the waiting time for public health care was too long. Some of the participants did not want to invest in shoes, although they acknowledged that saving on the cost of shoes was not wise.

R9: foot care is quite expensive nowadays. You can’t go there every day.

### Internal factors

#### Personality

Some personal traits had a negative effect on foot self-care activities. A lack of motivation also impaired foot self-care. The participants were not in the habit of looking after their feet; therefore, they did not do it frequently. However, the participants said they would like to have a daily foot self-care routine and they could see the importance of foot care and foot health. The participants acknowledged that their own lack of motivation led to not taking care of their feet. They often did not have the patience to carry out foot care; laziness also inhibited foot self-care. Some of them had no interest in moisturising and some simply did not remember to care for their feet. Feet are not on display as much as other parts of the body; therefore, little attention is paid to them. However, feelings of guilt and previous foot problems led to participants giving more thought to their feet.

R4: Well, unfortunately foot care is still quite modest, although it wouldn’t take more than fifteen minutes. I don’t understand why. Laziness.

R3: With these feet you just carry on until there’s a problem – then you have to be concerned.

#### Age-related characteristics

Ageing affects foot self-care activities. The participants admitted that they did not think about their feet when they were younger, and some had not altered their foot-care activities since then. The participants felt they could do less for themselves now that they were getting older. Foot care was seen as a necessity, however, and there was a need to seek help for foot care. Ageing was seen to slow down activity and increase health problems, and as they got older the participants noticed the importance of their feet.

R6: You don’t think when you’re younger. There are many good things that you don’t do. It didn’t even come to mind then. – It’s awful, but not much foot cream was used.

The participants described caring for their feet as part of looking after the lower extremities in general. Overall exercise was considered to be a necessity, and walking every day and doing physical exercise for the whole body were considered to be part of caring for the feet and keeping themselves functional. The participants were also concerned about other older people who were not active; for example, not walking regularly. Older age was seen as a promoting factor for respecting the body, and participants felt they needed to invest in themselves as they aged in order to maintain their functional ability for as long as possible.

## Discussion

In this article, foot self-care from the perspective of older people who were living at home, who were still active and who did not have any health issues that affected their feet, such as diabetes, were under investigation. Foot self-care was described as including basic foot-care activities. Our results revealed that changes in foot health and foot self-care cause anxiety in older people. The participants felt that their ability to look after their feet was limited by physical, external and internal factors. Moreover, older people considered the feet to be an intimate part of the body. They were willing to reveal their feet to health-care professionals for care purposes, but not to family members.

However, older people did not seek help from specialist foot-care providers as part of their overall health care; rather, they saw foot care as an indulgence and a pleasure. The feet play a major part in keeping active and maintaining functional ability. Therefore, maintaining one’s foot health should be considered as taking care of one’s overall wellbeing. This way of thinking can be ensured by changing attitudes towards feet and foot health in the health-care sector and the general population. However, older people are sometimes reluctant to seek professional help and they try to manage their foot problems themselves, and they think that a problem is severe enough to ask for help [39].

This research suggests that even though older people consider foot self-care to be important, they do not have established foot self-care routines and they need help and advice to find appropriate foot self-care practices, shoes and products. The participants noted that they did not receive advice about their feet unless they asked. Receiving foot care advice from a professional (e.g. a podiatrist) rather than family members builds confidence in foot self-care [40]. To enhance knowledge, habits and behaviour around foot self-care, there is a need to develop interventions for healthy older people who live at home.

Health-care professionals (e.g. registered nurses, nursing assistants and physicians) play a major role in promoting health and educating people about foot self-care. Previous studies [15, 41] found that older people needed partial or full assistance in washing the feet and cutting toenails, among other activities. Health-care professionals can help by caring for thickened toenails, noticing foot problems and referring older people to specialists [42]. However, these professionals need to be active in asking older people about their foot health. As highlighted in this study, older people consider the feet to be a private part of the body and they are willing to reveal them to and discuss them with health-care professionals only.

Increasing the awareness of foot self-care is important to promote health in older people. To achieve this empowering patient education is crucial to provide older people knowledge and demonstrate correct foot self-care practice. The patient education could be delivered in different forms ranging from face-to-face education to social media applications. Prevention and self-care are needed also due to rising health-care costs and people are living longer. Through education, it is possible to improve knowledge, confidence, and behaviours around foot self-care [10, 25, 36], and education leads to positive changes in foot health [9, 43]. It has been shown that most forms of education can have a positive effect [35, 44]. Health-care professionals are in a central position to support foot health and foot self-care in older people. Moreover, including foot health care in national level care guidelines and strategies would support the importance and awareness of it as recognised part of health care.

A contradictory finding in our study was that although older people seek professional help, they do not like to ask for help from their immediate family. For example, in diabetes self-care, social support has positively affected self-care behaviour [12, 45], however, people who are more likely to need help with foot care live alone [39]. It could be asked whether foot care is too intimate an issue to share with, for example, one’s own children. Health-care professionals could encourage people to ask for more support from their relatives and broach this intimate issue during appointments. In the UK, empowering older people and their carers to provide simple foot care is common practice [46, 47]. Practices from the UK could be adapted and implemented in the Finnish context.

Health-care professionals should consider the factors associated with foot self-care. Participants described ageing as affecting their ability to be active, and many of them described the need to do regular lower limb and foot exercises. Exercising can prevent, for example, expensive and life-changing falls. Ageing changes the characteristics of the feet [22], so people should be encouraged to do regular foot exercises to improve their balance. It has been shown that foot and ankle exercise programmes have influenced balance and ankle movement [48], and older people are more likely to keep to an exercise programme when they are supervised [49]. Similarly, footwear choices have been a contributing factor in falls and influence a person’s balance, walking confidence, and day-to-day activities [6]. The participants in this study did not usually wear shoes inside the home, as they felt that they were more likely to stumble when wearing shoes. With appropriate education, knowledge about types of shoes to wear indoors and outdoors can be increased and the related risks can be reduced. Although the participants had appropriate opinions about shoes that are good for their feet, they had difficulties in finding these types of shoes.

The results of this study can be used to develop education on foot self-care and the prevention of foot problems for older people. The participants described lacking motivation to care for their feet, only paying more attention to their feet when problems related to foot health have occurred. Prevention should start at an early stage (e.g. at school or, at the latest, during working life) to influence attitudes towards feet before any foot problems that affect functional ability or quality of life occur. This study supports the previous findings about health care professionals’ lack of attention to feet and foot problems [50]. There is a need to develop strategies and interventions increase knowledge of foot self-care and interest in feet. If feet are kept concealed and are not mentioned, they will remain an intimate part of the body and the importance of foot health will not be understood until foot problems cause difficulties. People are living longer and healthier lives today; by enhancing foot self-care older people can be empowered to stay active and reduce the financial burden of health care in societies.

Active involvement and responsibility of own health [51] are key issues also in foot self-care. Older people have foot problems which could be prevented or managed with proper foot self-care. The old generations have strong habits to behave according to guidance given by health care professionals. The foot self-care is often neglected and foot problems are left uncared. These issues pose several challenges for future health care and research to improve the recognition of foot health as part of general health. Future directions in clinical practice include assessing foot health and inquiring foot self-care activities in every health care contact, particularly in patients with long-term health conditions. This requires competence from health care professionals to identify foot problems, therefore regular in-service training to foot health care is recommended. For research purposes, cross-sectional studies with representative samples and valid instruments are needed to study frequency and activities used in foot self-care among older people. This approach could be reinforced with qualitative and observational research approaches to produce in-depth and focused information. Based on this information it is possible to plan intervention studies using for example the Behaviour Change Wheel [52] as framework to promote behaviour change in foot self-care in older people.

### Strengths and limitations

The process followed in this qualitative study was described by using the COnsolidated criteria for REporting Qualitative research, COREQ, [53] in order to maintain comprehensive reporting. The use of focus groups as a data collection method is considered suitable. In the groups, participants shared their opinions freely and willingly, although the rather shy participants might have benefitted from attending individual interviews. To add dependability to the study, participants who wished to receive the transcripts of their focus-group material were sent these by post for comments and/or corrections [32]. No additional comments were received. A co-author (TL) participated in all the focus groups to become familiar with the discussions and the participants. This increased the credibility of the study by enabling frequent debriefing sessions between researchers, peer scrutiny of the whole research process, and member checks of the data accuracy and analysis [54]. To maintain credibility, the co-authors evaluated the data analysis process, direct quotations, and analysis tables. Agreement on the appropriateness of category names and codes was achieved. There are limitations in the transferability of the results. The data was collected from a homogenous population in a relatively small town located in a sparsely populated northern country. Therefore, the results may not apply to all populations of older people. In addition, most of the study participants were older people who are active outside the home; information from people who tend to stay at home was not collected. However, the participants represented a range of ages and both genders; thus, a rich description of the phenomenon was achieved. No demographic data were asked for. Therefore, we do not know whether participants’ employment and educational background influenced their answers.

## Conclusion

These results are important for improving the welfare of older people and supporting their self-management as active citizens. This study adds the perspectives of older people on their feet and on foot self-care; this knowledge can be used in health care to enhance the prevention of foot problems. Future research is needed to support foot health in older people and improve their foot self-care activities.

## Abbreviations

COREQ: COnsolidated criteria for REporting Qualitative research
